# Development of a Care Bundle for Stroke Survivors with Psychological Symptoms: Evidence Summary and Delphi Study

**DOI:** 10.1155/2020/7836024

**Published:** 2020-06-29

**Authors:** Yiting Chen, Zheng Li, Jian Peng, Lanjun Shen, Juan Shi

**Affiliations:** ^1^School of Nursing, Fudan University, No. 305 Fenglin Road, Shanghai 200032, China; ^2^Shanghai Yangzhi Rehabilitation Hospital, No. 2209 Guangxing Road, Shanghai 201619, China

## Abstract

**Background:**

Psychological symptoms such as depression and anxiety are quite common among stroke survivors and have great negative impacts on patients.

**Objective:**

To develop a care bundle through reviewing and integrating care strategies for psychological symptoms after stroke and then improve the bundle by Delphi study.

**Methods:**

A structured search of the literature was performed to identify studies evaluating interventions for stroke patients with psychological symptoms such as depression and anxiety. Two trained researchers screened papers through the titles, abstracts, and full-texts independently. All studies complying with the eligibility criteria were appraised using quality assessment tools. Related interventions with evaluated evidence levels formed the preliminary bundle. Afterward, the Delphi study was carried out to improve the bundle, and the experts were contacted by e-mail. Ten clinical experts specialized in stroke and psychological rehabilitation were recruited. The reliability of experts was represented by the effective response rate and authority coefficient (Cr). The consensus was deemed to be reached when the mean score for item importance is all equal or above 3.50 and the coefficient of variation (CV) is all equal or below 0.20. The Kendall coefficient W test was adopted to evaluate the consensus on agreement among the experts as well. Data analysis was performed using SPSS V.22.0.

**Results:**

Through a systematic evidence summary and two-round Delphi study, the items that were given high scores and got consensus by experts were chosen for the bundle. The iDAME bundle consisted of five interventions eventually: maintaining **I**nteraction, tailored **D**iet, **A**cupressure, mindfulness **M**editation, and physical **E**xercise.

**Conclusion:**

The development of an evidence-based and consensus-based iDAME bundle which integrated western and traditional Chinese medicine intervention was described. Evidence summary made the bundle become scientific, while the Delphi study made it more maneuverable. Based on these results, the bundle would be potentially implemented in stroke patients for their psychological symptoms.

## 1. Introduction

Psychological symptoms are quite common among stroke survivors, such as depression, anxiety, apathy, and bipolar disorder [1]. An updated systematic review and meta-analysis found that the prevalence of poststroke depression was 31%∼33% [2], and 55% of patients might have depressive symptoms within 15 years after stroke [3]. The prevalence of anxiety within 10 years after stroke was 17%∼24%, and 57% of stroke patients might experience anxiety symptoms during this period [4]. It indicates that the adverse psychological symptoms of stroke patients occur in the long term and are persistent. Furthermore, psychological symptoms after stroke have great negative impacts on patients, such as self-efficacy reduction, rehabilitation stagnation, and much more heavy economic burden from the extended LOS (i.e., length of hospital stay) [5, 6].

At present, the management of psychological symptoms after stroke can be subdivided into pharmaceutical and nonpharmaceutical management. Common drugs used in clinical treatment include selective serotonin reuptake inhibitor (SSRI), serotonin-norepinephrine reuptake inhibitor (SNRI), a noradrenergic and specific serotonergic antidepressant (NaSSA), tricyclic antidepressants (TCAs), Wuling capsule (traditional Chinese medicine), and Shugan Jieyu capsule (traditional Chinese medicine) [7]. Though pharmaceutical treatments have a significant effect, most of them are accompanied by serious gastrointestinal reactions, liver or neurological damage, and other side effects. Current nonpharmaceutical management mainly includes physical therapy, psychotherapy, and traditional Chinese medicine treatment technology. Repetitive transcranial magnetic stimulation (rTMS) and transcranial direct current stimulation (tDCS) were applied to the treatment of psychological symptoms after stroke. A meta-analysis by Shen et al. analyzed a total of 1764 stroke patients in 22 randomized controlled trials and found that rTMS was effective for poststroke depression [8]. Bucur and Papagno demonstrated the efficacy and safety of tDCS and rTMS in stroke patients with psychological symptoms [9]. Exercise therapy such as muscle strengthening, mobilization exercise, and aerobic treadmill training was also confirmed that could provide positive contributions to mood improving after stroke through some clinical trials [10, 11]. Cognitive behavior therapy (CBT), behavioral activation therapy (BAT), problem-solving therapy (PST), mindfulness-based intervention (MBI), interpersonal therapy (IPT), and motivational interviewing (MI) are effective psychotherapies for mood disorders after stroke, suitable for patients of poor medication compliance and adverse drug reactions [7]. The effectiveness of traditional Chinese medicine treatment technology (e.g., acupuncture, moxibustion, and acupressure) and other adjuvant treatments such as music therapy, meditation, relaxation exercise, and mind-body exercise (e.g., Taichi, Qigong, and yoga) are also demonstrated and have the potential for clinical promotion [7, 12, 13]. However, the contributing factors of psychological symptoms are complex and various, and comprehensive interventions should be worked out. The existing combinations of interventions are empirical and lack of evidence-based. Hence, a synthesis of the existing literature to develop a care bundle for stroke patients with psychological symptoms is required.

A care bundle is a set of evidence-based or self-evident good-practice-based interventions for a defined patient population and care setting [14]. It is a concept proposed by the Institute for Healthcare Improvement (IHI), which refers to combining and integrating evidence-based treatments and nursing measures into a set of intervention program, so as to achieve the goal of treating clinical diseases and improve the quality of care and patients' outcomes [15]. The combination of multiple intervention measures is often more efficient and beneficial than the single performed alone.

In this study, we present the development of a poststroke psychological symptom care bundle called iDAME. iDAME is the abbreviation of maintaining **I**nteraction, tailored **D**iet, **A**cupressure, mindfulness **M**editation, and physical **E**xercise. These five interventions were obtained through evidence summary and two-round Delphi. The formation of the whole bundle is evidence-based; it not only combines the best available evidence but also attaches the importance to the individual clinical experience. The best evidence, practical experience, and patient's actual condition are comprehensively considered in medical decision making to achieve the transition from evidence to practice effectively.

## 2. Materials and Methods

### 2.1. Evidence Summary

#### 2.1.1. Literature Search

According to the 6S evidence resource pyramid model [16], related practical guidelines, evidence summaries, expert consensus, systematic reviews, and clinical trials for the management of poststroke psychological symptoms were retrieved.

To obtain empirical studies on the management of poststroke psychological symptoms, a comprehensive search of multiple databases from January 2010 to May 2019 was conducted, including SinoMed, Wan Fang Database, Chinese National Knowledge Infrastructure (CNKI), PubMed, EMBASE, CINAHL, Web of Science, JBI, and Cochrane (Table 1). In addition, the relevant clinical practice guideline websites were also searched, such as the Scottish Intercollegiate Guidelines Network (SIGN), the National Institute for Health and Care Excellence (NICE), the Registered Nurses' Association of Ontario, Canada (RNAO), American Heart Association/American Stroke Association (AHA/ASA), Neurocritical Care Society (NCS), European Stroke Organisation (ESO), Royal College of Physicians (RCP), National Stroke Foundation (NSF), and Royal Dutch Society for Physical Therapy (KNGF) using the search terms “stroke,” “cerebrovascular accident,” “psychological disorder,” “psychological symptom,” “mental problem,” “emotional problem,” and “mood stress.” Reference lists of relevant articles were cross-checked and pertinent journals were hand searched for articles. The inclusion criteria were (1) studies written in English and Chinese and (2) studies that involved psychological symptoms in the context of the poststroke period and addressed any aspect of care. The exclusion criteria were (1) duplicates, review articles, and unpublished manuscripts and (2) studies that did not report poststroke psychological symptoms in their findings.

#### 2.1.2. Search Results and Quality Appraisals

**Figure 1:**
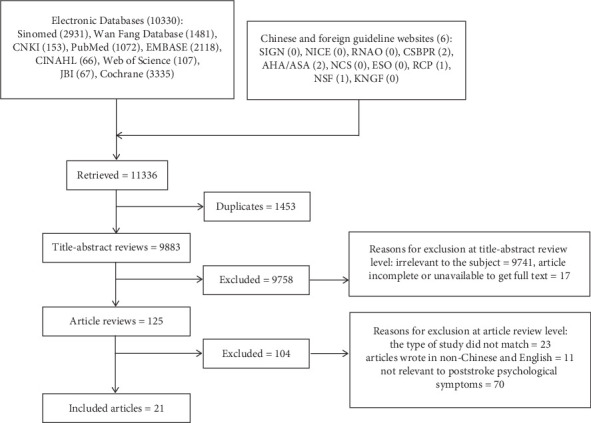
Flowchart of study selection.

Two trained authors (YTC&JP) screened the titles, abstracts, and full-texts independently. A total of 21 of the 11336 articles were included [7, 12, 13, 17–34] (Figure 1, Table 2). All studies meeting the eligibility criteria were appraised independently by three authors (YTC, JP, and LJS) through quality assessment tools (Table 3). The evaluation results are shown in Table 4.

#### 2.1.3. Interventions Extraction and the Preliminary Bundle

Evaluated evidence levels of related interventions were extracted by criteria of levels of evidence reported in the Canadian Best Practice Recommendations for Stroke Care [18]. The main interventions from 21 articles were extracted to form the preliminary bundle.

### 2.2. Delphi Study

A Delphi study was conducted to obtain experts' suggestions on the practicability and maneuverability of the bundle so as to improve the preliminary bundle further.

#### 2.2.1. Participants

The number of experts can vary according to the size and the scope of the research involved. Generally, it is advisable to be around 8–20 experts [38]. Considering that the scope of the whole research is specific (i.e., poststroke psychological management) and the interventions included in the preliminary bundle through the evidence summary were relatively common, it was proper to recruit ten clinical experts specialized in stroke and psychological rehabilitation.

#### 2.2.2. Delphi Survey Rounds

In the first round, each expert was told about the research question and provided with a three-part structured consulting questionnaire by e-mail. Based information of each expert was collected in part I, including age, gender, primary department, experience of dealing with psychological symptoms after stroke, and potential conflicts of interest. Part II included a list of interventions, and experts were asked to rate scores for the importance of the specific items (i.e., 56 items from the preliminary bundle interventions including “health education,” “emotion relieving,” “sleep improving,” “function promoting,” and “interaction maintaining”) using a Likert scale of 1–5 (1—Not important to 5—Very important). There were text boxes as well in Part II so that experts could write their comments about the interventions being considered. In part III, experts were asked to make an evaluation for the familiarity and judgment basis of the research questions. Two weeks after the e-mail delivery, an electronic reminder was sent by the researcher to those who had not yet completed the first round survey. Before the next round, some items were revised, excluded, or added based on the scores and opinions of experts in the first round. In the next round, updated consulting questionnaires (part II) were sent to experts by e-mail. The process was the same as the first round.

### 2.3. Data Analysis

All experts' based information and data in consulting questionnaires were sorted and recorded into Microsoft Excel firstly and then were performed using SPSS V.22.0 to generate descriptive statistics.

The reliability of experts is usually represented by the effective response rate and authority coefficient (Cr) [39]. Cr is determined by familiarity (Cs) and judgment basis (Ca) of the research questions [Cr = (Cs + Ca)/2]. Cs represents experts' familiarity with the research question, and a Likert scale of 0.1, 0.3, 0.5, 0.7, and 0.9 (0.1—Not familiar to 0.9—Very familiar) was used. Ca represents the judgment criteria the experts are based on. The criteria's influence degrees and score assignments are theoretical analysis (great-0.5; medium-0.4; small-0.3), working experience (great-0.2, medium-0.2, small-0.1), referring to literature (great-0.2, medium-0.1, small-0.1) and self-intuition (great-0.1, medium-0.1, small-0.1). Ca value is the sum score of these criteria.

The importance of the specific items in the consulting questionnaire was evaluated by experts using a Likert scale of 1–5 (1–Not important to 5–Very important). The average rate score is presented as mean ± standard deviation (SD), and coefficient of variation (CV) defined as the SD divided by the mean is used to describe the relative dispersion degree of the items' importance evaluation from experts [40]. The lower the CV value, the higher the coordination degree of experts' opinions. There have been no universally accepted criteria for consensus in a Delphi study [41–43]. This study defines that when the mean score for the importance of item is all equal or above 3.50 and CV is all equal or below 0.20, the consensus is reached [44]. In other words, the Delphi survey can finish when all items in the consulting questionnaire are met the two criteria above. The Kendall coefficient *W* test was adopted to evaluate the consensus on agreement among the experts as well. A two-tailed *p* value of <0.05 was considered statistically significant.

## 3. Results

### 3.1. The Reliability of Experts

The age of experts was 43.10 ± 6.28 years old, and the length of working in this field was 17.80 ± 7.38 years. All experts had no conflicts of interest. The effective response rates of the 2 rounds were 100%. The mean value of the expert authority coefficient (Cr) was 0.85 (Table 5). The authority of the experts in this study was relatively high, and the results were thus trustworthy.

### 3.2. The First Round

Of the 56 items rated by experts in the first round, one item's mean score and seventeen items' CVs did not meet the criteria (Table 6). The relative dispersion degree of the items' importance evaluation from experts was high. Kendall's coefficient of concordance (*W*) was calculated to be 0.31 (*p* < 0.01). What's more, some experts suggested that the structure of the questionnaire should be adjusted so as to be more clear for clinical professionals and patients to understand and implement in the future. A care bundle is a set of self-evident good-practice-based interventions for a defined patient population and care setting. It was generally a set of three to five evidence-based practices and there was no need to reach every aspect. Therefore, the questionnaire was greatly modified. The main five interventions were changed from “health education,” “emotion relieving,” “sleep improving,” “function promoting,” and “interaction maintaining” to “tailored diet,” “acupressure,” “mindfulness meditation,” “physical exercise,” and “maintaining interaction.” Eighteen existing items were merged according to the experts' comments (e.g., health education was incorporated into maintaining interaction actually). Ten items such as “medication education” and “color emotion management” were deleted because of great heterogeneity in experts' rate scores (i.e., CV value). And one item (i.e., home visits per month) was deleted because it is unavailable for professionals to have home visits for patients who lived in different provinces far away from the hospital. A new expert-proposed item was added. Recreation rehabilitation such as playing chess or cards, reading newspapers, and playing games was proposed to be added by some experts. Though there was no high-quality evidence, the experts had concluded that there was a positive effect on improving the bad mood for stroke patients from their own experiences. Therefore, it was included additionally. As a result, the revised consulting questionnaire contained five interventions and 28 items before the second round.

### 3.3. The Second Round

The results showed that all mean scores for importance on items were all above 3.50 and CVs were all 0.20 or below in the second round (Table 6). Because the structure of the questionnaire changed a lot, Kendall's coefficient of concordance (*W*) decreased to 0.28 (*p* < 0.01). But it was statistically significant. There was no additional comment offered by experts in this round. The consensus was reached, and the Delphi survey was finished. The bundle was improved and finalized. Five interventions (i.e., maintaining Interaction, tailored Diet, Acupressure, mindfulness Meditation, and physical Exercise) made up the iDAME bundle eventually.

## 4. Discussion

A care bundle integrated western and traditional Chinese medicine interventions together for stroke patients with psychological symptoms were successfully developed. Previous studies showed that each intervention of the iDAME care bundle could improve the negative emotions after strokes, such as depression and anxiety indeed. Through the two-round Delphi, the maneuverability of iDAME care bundle has been approved further.

As western humoralism considering human body was retained to be a container of four liquids (blood, phlegm, black bile, and yellow bile), and health was constituted by the state of equilibrium of these substances, traditional Chinese medicine (TCM) holds that health depends on the balance of Qi, blood, Yin, and Yang in the human body [45, 46]. Different states of Qi, blood, Yin, and Yang make people have different body constitutions (“Tizhi” in Chinese). Common classifications of TCM constitutions are neutral constitution, Qi-deficiency constitution, Yang-deficiency constitution, Yin-deficiency constitution, phlegm-damp constitution, damp-heat constitution, blood-stagnation constitution, Qi-stagnation constitution, and special diathesis constitution [47]. According to TCM constitution theory, all constitutions except neutral constitution (balanced constitution) mean disharmony and can be viewed as an individual's susceptibility to specific disease or symptoms [48]. TCM believes that the constitution is partly genetically determined and partly acquired, and classifies individuals' constitution into the above nine types based on Chinese medical theory, multidisciplinary studies, and clinical practice. Hence, the results can be used in disease treatment and rehabilitative care [46, 49, 50]. TCM constitution is a comprehensive and relatively stable trait in the aspects of morphological structure, physiological function, and psychological state, which determines an individual's susceptibility to a certain pathogenic factor and its tendency of pathological changes [47]. Various types of constitution show various characteristics. For example, patients of stagnant Qi constitution are mostly thin and often feel gloomy or depressed, easy to be nervous, anxious, and sensitive [49]. Patients with different constitutions require tailored care for the prevention and cure of poststroke psychological symptoms. Therefore, the iDAME care bundle includes TCM constitution theory in part of interventions, and patients with different constitutions are given specific care.

Nutrients are essential for the optimal production of neurotransmitters affecting mood [51]. Mediterranean diet pattern and other nutrient elements included in the bundle (e.g., deep-sea fishes rich in *n* − 3 polyunsaturated fatty acid) have been proved to be beneficial to the improvement of negative emotions [23]. Due to diverse TCM constitutions, the bundle offers choices to different patients. For instance, patients of the Qi-stagnation constitution are encouraged to feed wheat, oranges, radishes, and so on. Sleep disturbances are frequently reported in stroke patients and associated with the mood state after strokes [52]. Hence, insomnia patients are given a sleeping-improving diet to improve sleep quality according to their TCM constitutions. For example, insomniac patients of the Yin-deficiency constitution are suggested to drink some milk and honey water, while patients of the Yang-deficiency constitution are suggested to eat some walnuts and dried longans. An individualized diet helps patients adjust negative emotions better.

Acupressure is one of the Complementary and Alternative Medicine (CAM) modalities, which has the potential for promoting sleep quality and psychological wellbeing [53]. It is a stimulation of acupuncture points using fingers, palms, or devices to balance body energy, maintain good health, and prevent illness [54]. Acupoint stimulation regulates the autonomic nervous system by reducing sympathetic activities and increasing parasympathetic activity, which can reduce the stress response and induce relaxation. Regulating the autonomic nervous system, hormonal factors and neurotransmitters may have biological effects on inducing sleep, calmness, and feelings of psychological wellness [53]. Common acupuncture points for stroke patients with bad mood are Shaohai, Kunlun, Taichong, and so on. The bundle offers additional choices to different TCM constitution patients (e.g., Guanyuan, Qihai, and Shenque for Qi-deficiency patients) as well. Given that music engages in a variety of brain areas involved in emotion, musical interventions have been used to solve mental problems [55]. Moreover, music helps patients get into a relaxed state more easily, so specific pieces of music are supplied to patients during acupressure (e.g., Mozart's Serenade).

Mindfulness meditation can be defined as a form of spiritual training that aims to improve core psychological capacities, such as attentional and emotional self-regulation [56]. Improvements in emotion regulation associated with mindfulness meditation have been investigated through various approaches [57]. Mindfulness meditation works by strengthening prefrontal cognitive control mechanisms and thus downregulates activity in regions relevant to affect processing, such as the amygdala [56]. Stroke patients with psychological symptoms calm down and focus on what they are doing better.

Physical exercise is indispensable. Studies showed that appropriate exercise could improve mental health status and prevent mechanisms that mediate the association between depression and metabolic syndrome [12, 58–60].

Last but not least, maintaining interaction is an important part of this bundle, which makes patients and caregivers solve the problems more easily with help from medical professionals. They can talk with nurses or ward mates to relieve stress and emotions through social software (Wechat texts and videos chatting, group chatting, etc.). Medical professionals could provide information supporting system for patients and caregivers. It is also the guarantee of the entire bundle's accomplishment after discharge. Patients will be reminded of completing interventions and be helped when they are in trouble. The use of a network platform can improve the compliance of patients [61].

iDAME care bundle is an entirety including physical and psychological interventions for the poor mood of stroke patients. The whole bundle is relatively simple and practical. However, there are still some limitations. Firstly, the quality of evidence included is not pretty high, there is a risk of bias to some extent. Secondly, the whole bundle is content-rich, integrating Chinese and western interventions, so the compliance of the patient is unknown. Thirdly, though each intervention of the bundle has been shown to be effective in improving the psychological symptoms after stroke, the combination effect has yet to be verified.

## 5. Conclusions

iDAME bundle was developed through a systematic evidence summary and two-round Delphi study. It is an entirety including physical and psychological interventions. The scientificity of iDAME bundle has been approved. Furthermore, an implementation evaluation is required to confirm the feasibility of clinical.

## Figures and Tables

**Table 1:** The search terms in English.

JBI	#1 Stroke or hemiplegia or “cerebrovascular disease” or “cerebral arterial thrombosis” or “Ischemic stroke”
	#2 depression or anxiety
	#3 yr = “2010-Current”
	#1 and #2 and #3

Cochrane	#1 Stroke or strokes or hemiplegia or “brain infarction” or “cerebrovascular disease” or CVD or “cerebrovascular attack” or “cerebrovascular accident” or “cerebrovascular accidents” or CVA or “brain vascular accident” or “brain vascular accidents” or “acute cerebrovascular accident” or “cerebrovascular apoplexy” or “cerebrovascular stroke” or “cerebrovascular strokes” or “acute stroke” or “acute strokes” or “cerebral strokes” or “cerebral stroke” or “cerebral arterial thrombosis” or “Ischemic stroke”
	#2 Psychological or Psychology or “psychological disorders” or “psychological disorder” or “psychological stress” or “psychological health” or “Psychological symptom” or “Psychological symptoms” or “psychological problem” or “psychological problems” or mental or “mental health” or “mental symptoms” or “mental symptom” or “mental disorder” or “mental disorders” or “mental problem” or “mental problems” or “mental stress” or emotional or emotion or “emotional stress” or “emotional symptoms” or “emotional symptom” or “emotional disorder” or “emotional disorders” or “emotional problem” or “emotional problems” or “emotional health” or mood or “mood disorders” or “mood disorder” or “mood health” or “mood stress” or “mood symptom” or “mood symptoms” or “mood problem” or “mood problems” or affectivity or affective or “affective disorder” or “affective disorders” or “affective health” or “affective stress” or “affective problem” or “affective problems” or “affective symptom” or “affective symptoms” or depression or “depression symptom” or “depression symptoms” or “depression disorder” or “depression disorders” or anxiety or “anxiety symptom” or “anxiety symptoms” or “anxiety disorder” or “anxiety disorders”
	#3 “Randomized controlled trial ” or ”Randomized controlled trials” or RCT or RCTs or “systematic review” or “systematic reviews” or meta-analysis or guideline or guidelines or consensus
	#4 2010-current
	#1 and #2 and #3 and #4

Pubmed	#1 Stroke[Title/Abstract] OR strokes[Title/Abstract] OR hemiplegia[Title/Abstract] OR “brain infarction” [Title/Abstract] OR “cerebrovascular disease” [Title/Abstract] OR CVD[Title/Abstract] OR “cerebrovascular attack” [Title/Abstract] OR “cerebrovascular accident” [Title/Abstract] OR “cerebrovascular accidents” [Title/Abstract] OR CVA[Title/Abstract] OR “brain vascular accident” [Title/Abstract] OR “brain vascular accidents” [Title/Abstract] OR “acute cerebrovascular accident”[Title/Abstract] OR “cerebrovascular apoplexy”[Title/Abstract] OR “cerebrovascular stroke” [Title/Abstract] OR “cerebrovascular strokes” [Title/Abstract] OR “acute stroke” [Title/Abstract] OR “acute strokes” [Title/Abstract] OR “cerebral strokes” [Title/Abstract] OR “cerebral stroke” [Title/Abstract] OR “cerebral arterial thrombosis” [Title/Abstract] OR “Ischemic stroke” [Title/Abstract]
	#2 Psychological[Title/Abstract] OR Psychology[Title/Abstract] OR “psychological disorders” [Title/Abstract] OR “psychological disorder” [Title/Abstract] OR “psychological stress” [Title/Abstract] OR “psychological health”[Title/Abstract] OR “Psychological symptom” [Title/Abstract] OR “Psychological symptoms” [Title/Abstract] OR “psychological problem” [Title/Abstract] OR “psychological problems” [Title/Abstract] OR mental[Title/Abstract] OR “mental health” [Title/Abstract] OR “mental symptoms” [Title/Abstract] OR “mental symptom” [Title/Abstract] OR “mental disorder” [Title/Abstract] OR “mental disorders” [Title/Abstract] OR “mental problem” [Title/Abstract] OR “mental problems “ [Title/Abstract] OR “mental stress” [Title/Abstract] OR emotional[Title/Abstract] OR emotion[Title/Abstract] OR “emotional stress” [Title/Abstract] OR “emotional symptoms” [Title/Abstract] OR “emotional symptom” [Title/Abstract] OR “emotional disorder” [Title/Abstract] OR “emotional disorders” [Title/Abstract] OR “emotional problem” [Title/Abstract] OR “emotional problems” [Title/Abstract] OR “emotional health” [Title/Abstract] OR mood[Title/Abstract] OR “mood disorders” [Title/Abstract] OR “mood disorder” [Title/Abstract] OR “mood health” [Title/Abstract] OR “mood stress” [Title/Abstract] OR “mood symptom” [Title/Abstract] OR “mood symptoms” [Title/Abstract] OR “mood problem” [Title/Abstract] OR “mood problems” [Title/Abstract] OR affectivity[Title/Abstract] OR affective[Title/Abstract] OR “affective disorder” [Title/Abstract] OR “affective disorders” [Title/Abstract] OR “affective health” [Title/Abstract] OR “affective stress” [Title/Abstract] OR “affective problem” [Title/Abstract] OR “affective problems” [Title/Abstract] OR “affective symptom” [Title/Abstract] OR “affective symptoms” [Title/Abstract] OR depression[Title/Abstract] OR “depression symptom” [Title/Abstract] OR “depression symptoms” [Title/Abstract] OR “depression disorder” [Title/Abstract] OR “depression disorders” [Title/Abstract] OR anxiety[Title/Abstract] OR “anxiety symptom” [Title/Abstract] OR “anxiety symptoms” [Title/Abstract] OR “anxiety disorder” [Title/Abstract] OR “anxiety disorders” [Title/Abstract]
	#3 ”Randomized controlled trial ” [Title/Abstract] OR “Randomized controlled trials” [Title/Abstract] OR RCT[Title/Abstract] OR RCTs[Title/Abstract] OR “systematic review” [Title/Abstract] OR “systematic reviews” [Title/Abstract] OR meta-analysis[Title/Abstract] OR guideline[Title/Abstract] OR guidelines[Title/Abstract] OR consensus[Title/Abstract])
	#4 Publication date from 2010/01/01
	#1 and #2 and #3 and #4

Web of science	#1 TITLE : Stroke or strokes or hemiplegia or “brain infarction” or “cerebrovascular disease” or CVD or “cerebrovascular attack” or “cerebrovascular accident” or “cerebrovascular accidents” or CVA or “brain vascular accident” or “brain vascular accidents” or “acute cerebrovascular accident” or “cerebrovascular apoplexy” or “cerebrovascular stroke” or “cerebrovascular strokes” or “acute stroke” or “acute strokes” or “cerebral strokes” or “cerebral stroke” or “cerebral arterial thrombosis” or “Ischemic stroke”
	#2 TITLE : Psychological or Psychology or “psychological disorders” or “psychological disorder” or “psychological stress” or “psychological health” or “Psychological symptom” or “Psychological symptoms” or “psychological problem” or “psychological problems” or mental or “mental health” or “mental symptoms” or “mental symptom” or “mental disorder” or “mental disorders” or “mental problem” or “mental problems” or “mental stress” or emotional or emotion or “emotional stress” or “emotional symptoms” or “emotional symptom” or “emotional disorder” or “emotional disorders” or “emotional problem” or “emotional problems” or “emotional health” or mood or “mood disorders” or “mood disorder” or “mood health” or “mood stress” or “mood symptom” or “mood symptoms” or “mood problem” or “mood problems” or affectivity or affective or “affective disorder” or “affective disorders” or “affective health” or “affective stress” or “affective problem” or “affective problems” or “affective symptom” or “affective symptoms” or depression or “depression symptom” or “depression symptoms” or “depression disorder” or “depression disorders” or anxiety or “anxiety symptom” or “anxiety symptoms” or “anxiety disorder” or “anxiety disorders”
	#3 TITLE: “Randomized controlled trial ” or ”Randomized controlled trials” or RCT or RCTs or “systematic review” or “systematic reviews” or meta-analysis or guideline or guidelines or consensus
	#4 Timespan: 2010-2019. Indexes: SCI-EXPANDED, SSCI, A&HCI, CPCI-S, CPCI-SSH, ESCI, CCR-EXPANDED, IC
	#1 and #2 and #3 and #4

Embase	#1 Stroke or hemiplegia or “cerebrovascular disease” or “cerebral arterial thrombosis” or “Ischemic stroke”
	#2 depression or anxiety
	#3 yr = “2010”
	#1 and #2 and #3

CINAHL	#1 TI ( Stroke or strokes or hemiplegia or “brain infarction” or “cerebrovascular disease” or CVD or “cerebrovascular attack” or “cerebrovascular accident” or “cerebrovascular accidents” or CVA or “brain vascular accident” or “brain vascular accidents” or “acute cerebrovascular accident” or “cerebrovascular apoplexy” or “cerebrovascular stroke” or “cerebrovascular strokes” or “acute stroke” or “acute strokes” or “cerebral strokes” or “cerebral stroke” or “cerebral arterial thrombosis” or “Ischemic stroke”
	#2 TI ( Psychological or Psychology or “psychological disorders” or “psychological disorder” or “psychological stress” or “psychological health” or “Psychological symptom” or “Psychological symptoms” or “psychological problem” or “psychological problems” or mental or “mental health” or “mental symptoms” or “mental symptom” or “mental disorder” or “mental disorders” or “mental problem” or “mental problems” or “mental stress” or emotional or emotion or “emotional stress” or “emotional symptoms” or “emotional symptom” or “emotional disorder” or “emotional disorders” or “emotional problem” or “emotional problems” or “emotional health” or mood or “mood disorders” or “mood disorder” or “mood health” or “mood stress” or “mood symptom” or “mood symptoms” or “mood problem” or “mood problems” or affectivity or affective or “affective disorder” or “affective disorders” or “affective health” or “affective stress” or “affective problem” or “affective problems” or “affective symptom” or “affective symptoms” or depression or “depression symptom” or “depression symptoms” or “depression disorder” or “depression disorders” or anxiety or “anxiety symptom” or “anxiety symptoms” or “anxiety disorder” or “anxiety disorders”
	#3 AB (“Randomized controlled trial” or ”Randomized controlled trials” or RCT or RCTs or “systematic review” or “systematic reviews” or meta-analysis or guideline or guidelines or consensus)
	#4 2010–2019
	#1 and #2 and #3 and #4

**Table 2:** Study characteristics arranged by type of evidence.

Year	Author/organization	Title	Type of the evidence
2018	ASA/AHA	Guidelines for the early management of patients with acute ischemic stroke [20]	Guideline
2017	NSF	Clinical guidelines for stroke management [19]	Guideline
2016	CSBPR	Managing transitions of care following stroke [17]	Guideline
2016	ASA/AHA	Guidelines for adult stroke rehabilitation and recovery [22]	Guideline
2016	RCP	National clinical guideline for stroke [21]	Guideline
2015	CSBPR	Mood, cognition and fatigue following stroke practice guidelines [18]	Guideline
2016	CMDA	Chinese expert consensus on the clinical practice of post-stroke depression [7]	Expert consensus
2019	JBI	Depression in stroke: exercise [12]	Evidence summary
2017	JBI	Post-stroke depression: management [13]	Evidence summary
2019	Firth et al.	The effects of dietary improvement on symptoms of depression and anxiety: a meta-analysis of randomized controlled trials [23]	Systematic review
2018	Waits et al.	Acupressure effect on sleep quality: a systematic review and meta-analysis [24]	Systematic review
2018	Zou et al.	Baduanjin exercise for stroke rehabilitation: a systematic review with meta-analysis of randomized controlled trials [25]	Systematic review
2018	Zou et al.	Effects of mind-body exercises for mood and functional capabilities in patients with stroke: an analytical review of randomized controlled trials [26]	Systematic review
2018	Lyu et al.	Tai chi for stroke rehabilitation: a systematic review and meta-analysis of randomized controlled trials [27]	Systematic review
2015	Au et al.	Effects of acupressure on anxiety: a systematic review and meta-analysis [28]	Systematic review
2014	Goyal et al.	Meditation programs for psychological stress and well-being: a systematic review and meta-analysis [29]	Systematic review
2019	Le Danseur et al.	Music as a therapy to alleviate anxiety during inpatient rehabilitation for stroke [30]	Randomized controlled trial
2017	Vahlberg et al.	Short-term and long-term effects of progressive resistance and balance exercise program in individuals with chronic stroke: a randomized controlled trial [31]	Randomized controlled trial
2012	Johansson et al.	Mindfulness-based stress reduction (MBSR) improves long-term mental fatigue after a stroke or traumatic brain injury [32]	Randomized controlled trial
2015	Zhi et al.	Analysis of distribution characteristics of TCM constitution types in post-stroke depression patients [33]	Cross-sectional study
2013	Verdonschot et al.	Symptoms of anxiety and depression assessed with the hospital anxiety and depression scale in patients with oropharyngeal dysphagia [34]	Cross-sectional study

**Table 3:** Quality assessment tools.

Type of study	Quality assessment tools	Contains	Method (details)
Guideline	Appraisal of guidelines for research and evaluation (AGREE II) [35]	6 domains 23 items 2 additional items of the overall assessment	Each item is rated on a 7-point scale (1—strongly disagree to 7—strongly agree). And a quality score is calculated for each of the six domains. Scores of the six domains are independent and could not be aggregated into one score. Each domain score is the sum of all item scores (obtained score). The calculating method of scaled domain score is (obtained score – minimum possible score)/(maximum possible score – minimum possible score). Maximum possible score = 7 (strongly agree) ∗ number of items *∗* number of appraisers, minimum possible score = 1 (strongly disagree) *∗* number of items ∗ number of appraisers. Two additional items of overall assessment are graded on a scale of 1 (lowest possible quality) to 7 (highest possible quality).

Expert consensus	The authenticity evaluation method of consensus articles of the Joanna Briggs Institute [36]	6 items	Each item is evaluated by Yes, No, Unclear, or Not Applicable.

Evidence summary	The quality assessment tools of guidelines, systematic reviews, and original studies included	—	—

Systematic review	Assessment of Multiple Systematic Reviews 2 (AMASTAR2) [37]	16 items	Each item is evaluated by Yes, Partial Yes, and No.

Randomized controlled trial	The authenticity evaluation method of RCT articles of the Joanna Briggs Institute [36]	13 items	Each item is evaluated by Yes, No, Unclear, or Not Applicable.

Cross-sectional study	The authenticity evaluation method of cross-sectional articles of the Joanna Briggs Institute [36]	8 items	Each item is evaluated by Yes, No, Unclear, or Not Applicable.

**(a):** 

Guideline	Scope and purpose	Stakeholder involvement	Rigour of development	Clarity of presentation	Applicability	Editorial independence	Numbers of domain (≥60%)	Numbers of domain (≥30%)
ASA/AHA, 2018	65.28	56.94	80.21	91.67	28.13	83.33	4	5
NSF, 2017	87.50	95.83	81.77	83.33	69.79	93.75	6	6
CSBPR, 2016	75.93	72.22	67.36	90.74	56.94	100	5	6
ASA/AHA, 2016	77.78	62.96	41.67	74.07	31.94	100	4	6
RCP, 2016	88.89	86.11	80.21	83.33	59.38	97.92	5	6
CSBPR, 2015	75.93	72.22	67.36	90.74	56.94	100	5	6

**(b):** 

Items (expert consensus)	Evaluation (yes/no/unclear/not applicable)
Is the source of the opinion clearly identified?	Yes
Does the source of opinion have standing in the field of expertise?	Yes
Are the interests of the relevant population the central focus of the opinion?	Yes
Is the stated position the result of an analytical process, and is there logic in the opinion expressed?	Yes
Is there reference to the extant literature?	Yes
Is any incongruence with the literature/sources logically defended?	No

**(c):** 

Items (systematic review)	Eng^*∗*^	Saunders^*∗*^	Graven^*∗*^	Ginkel^*∗*^	Hackett^*∗*^	Firth	Goyal	Lyu	Zou	Zou	Au	Waits
Did the research questions and inclusion criteria for the review include the components of PICO?	Yes	Yes	Yes	No	Yes	Yes	Yes	Yes	Yes	Yes	Yes	Yes
Did the report of the review contain an explicit statement that the review methods were established prior to the conduct of the review and did the report justify any significant deviations from the protocol?	No	Yes	No	No	Yes	Yes	No	Yes	Yes	No	No	No
Did the review authors explain their selection of the study designs for inclusion in the review?	Yes	Yes	Yes	No	Yes	Yes	Yes	Yes	Yes	Yes	No	No
Did the review authors use a comprehensive literature search strategy?	Yes	Yes	Partial Yes	Partial Yes	Yes	Yes	Partial yes	Partial yes	Partial yes	Partial yes	Partial yes	Yes
Did the review authors perform study selection in duplicate?	Yes	Yes	Yes	No	Yes	Yes	Yes	Yes	Yes	Yes	Yes	Yes
Did the review authors perform data extraction in duplicate?	Yes	Yes	No	No	Yes	No	Yes	Yes	Yes	Yes	Yes	Yes
Did the review authors provide a list of excluded studies and justify the exclusions?	No	Yes	No	No	Yes	Yes	No	No	No	No	No	No
Did the review authors describe the included studies in adequate detail?	Yes	Yes	Yes	Yes	Yes	Yes	Yes	Yes	Yes	Yes	No	Yes
Did the review authors use a satisfactory technique for assessing the risk of bias (RoB) in individual studies that were included in the review?	Yes	Yes	Yes	No	Yes	Partial Yes	Yes	Yes	Yes	Yes	Yes	Yes
Did the review authors report on the sources of funding for the studies included in the review?	No	No	No	No	No	No	No	No	No	No	No	No
If meta-analysis was performed, did the review authors use appropriate methods for statistical combination of results?	Yes	Yes	Yes	No meta-analysis	Yes	Yes	Yes	Yes	Yes	Yes	Yes	Yes
If meta-analysis was performed, did the review authors assess the potential impact of RoB in individual studies on the results of the meta-analysis or other evidence synthesis?	Yes	Yes	Yes	No meta-analysis	Yes	Yes	Yes	Yes	Yes	Yes	Yes	Yes
Did the review authors account for RoB in individual studies when interpreting/discussing the results of the review?	Yes	Yes	Yes	No	Yes	Yes	Yes	Yes	Yes	Yes	Yes	Yes
Did the review authors provide a satisfactory explanation for, and discussion of, any heterogeneity observed in the results of the review?	Yes	Yes	Yes	No	Yes	Yes	Yes	Yes	Yes	Yes	Yes	Yes
If they performed quantitative synthesis did the review authors carry out an adequate investigation of publication bias (small study bias) and discuss its likely impact on the results of the review?	No	Yes	No	No	Yes	Yes	Yes	No	No	No	No	No
Did the review authors report any potential sources of conflict of interest, including any funding they received for conducting the review?	Yes	Yes	No	Yes	Yes	No	Yes	Yes	Yes	Yes	Yes	Yes

^*∗*^These five systematic reviews are mentioned in the evidence summary of JBI.

**(d):** 

Items (randomized controlled trial)	Vahlberg	Johansson	Danseur
Was true randomization used for assignment of participants to treatment groups?	Yes	Unclear	Yes
Was allocation to treatment groups concealed?	Yes	No	Yes
Were treatment groups similar at the baseline?	Unclear	Yes	Yes
Were participants blind to treatment assignment?	No	Not Applicable	Not Applicable
Were those delivering treatment blind to treatment assignment?	No	Not Applicable	Not Applicable
Were outcomes assessors blind to treatment assignment?	Yes	No	No
Were treatment groups treated identically other than the intervention of interest?	Yes	Yes	Yes
Was follow-up complete and if not, were differences between groups in terms of their follow-up adequately described and analyzed?	Yes	No	No
Were participants analyzed in the groups to which they were randomized?	Yes	No	No
Were outcomes measured in the same way for treatment groups?	Yes	Yes	Yes
Were outcomes measured in a reliable way?	Yes	Yes	Yes
Was appropriate statistical analysis used?	Yes	Yes	Yes
Was the trial design appropriate, and any deviations from the standard RCT design (individual randomization, parallel groups) accounted for in the conduct and analysis of the trial?	Yes	Yes	Yes

**(e):** 

Items (cross-sectional study)	Verdonschot	Zhi
Were the criteria for inclusion in the sample clearly defined?	Yes	Yes
Were the study subjects and the setting described in detail?	Yes	Yes
Was the exposure measured in a valid and reliable way?	Yes	Yes
Were objective, standard criteria used for measurement of the condition?	Yes	Yes
Were confounding factors identified?	Yes	No
Were strategies to deal with confounding factors stated?	Yes	No
Were the outcomes measured in a valid and reliable way?	Yes	Yes
Was appropriate statistical analysis used?	Yes	Yes

**Table 5:** The reliability of experts (Ca, Cs, and Cr).

Expert number	Criterion				Score		
	Theoretical analysis	Working experience	Referring to literature	Self-intuition	Ca	Cs	Cr
1	0.5	0.2	0.2	0.1	1.0	0.7	0.85
2	0.4	0.2	0.2	0.1	0.9	0.9	0.90
3	0.5	0.2	0.2	0.1	1.0	0.7	0.85
4	0.5	0.2	0.2	0.1	1.0	0.5	0.75
5	0.5	0.2	0.2	0.1	1.0	0.7	0.85
6	0.5	0.2	0.2	0.1	1.0	0.5	0.75
7	0.5	0.2	0.2	0.1	1.0	0.9	0.95
8	0.5	0.2	0.2	0.1	1.0	0.7	0.85
9	0.5	0.2	0.2	0.1	1.0	0.7	0.85
10	0.5	0.2	0.2	0.1	1.0	0.7	0.85

**Table 6:** Items and evidence levels of Round 1 and Round 2 items.

Items	Rating of importance (means ± SDs, CV)	
	Round 1	Round 2
1. Health education	5.00 ± 0.00, 0.00	Merged
1.1. Evaluating patients and caregivers, and providing health education about management of poststroke psychological symptoms (level B)	4.80 ± 0.63, 0.13	Merged
① Explaining the relationship between TCM constitution and symptoms	3.10 ± 1.20, 0.39	Merged
② Explaining basic mechanism of symptoms	4.10 ± 0.74, 0.18	Merged
③ Elaborating relevant factors of symptoms	4.40 ± 0.70, 0.16	Merged
④ Listing manifestations of symptoms	4.60 ± 0.52, 0.11	Merged
⑤ Listing negative outcomes of symptoms	4.80 ± 0.42, 0.09	Merged
⑥ Emphasize the importance of management	4.60 ± 0.70, 0.15	Merged

2. Emotion relieving	4.90 ± 0.32, 0.07	Merged
2.1. Tailored diet	4.50 ± 0.85, 0.19	4.40 ± 0.70, 0.16
① Eating mood-improving foods (level A)	4.00 ± 1.15, 0.29	4.20 ± 0.63, 0.15
② Mediterranean dietary pattern (level C)	3.80 ± 1.03, 0.27	4.10 ± 0.74, 0.18
③ Proper diet according to the patient's traditional Chinese medicine (TCM) constitution (level C)	3.70 ± 0.95, 0.26	3.70 ± 0.68, 0.18
④ Insomnia patients add Chinese medicine sleeping-improving diet according to patient's TCM constitution (level C)	-	4.70 ± 0.48, 0.10
2.2. Medication education (level C)	4.60 ± 0.70, 0.15	Deleted
① Antihypertensive drugs	4.30 ± 0.95, 0.22	Deleted
② Hypolipidemic drugs	4.10 ± 0.88, 0.21	Deleted
③ Antiplatelet drugs	4.20 ± 0.92, 0.22	Deleted
④ Anticoagulant drugs	4.30 ± 0.95, 0.22	Deleted
⑤ Neurotrophic drugs	3.60 ± 1.08, 0.30	Deleted
⑥ Mood-improving drugs	4.40 ± 0.70, 0.16	Deleted
2.3. Acupressure	3.90 ± 0.74, 0.19	4.00 ± 0.67, 0.17
① Pressing relevant fixed points (level A)	4.00 ± 0.67, 0.17	3.90 ± 0.57, 0.15
② Adding other acupressure points according to patient's TCM constitution (level C)	4.10 ± 0.57, 0.14	4.00 ± 0.67, 0.17
③ Insomnia patients press hypnotic points before sleeping (level B)	-	4.10 ± 0.74, 0.18
④ Each point for 3 minutes twice a day (level A)	-	4.00 ± 0.67, 0.17
⑤ Listening to soothing music while having acupressure (level A)	-	4.40 ± 0.52, 0.12
2.4. Mindfulness meditation	4.10 ± 0.57, 0.14	4.20 ± 0.42, 0.10
① Comfortable body positions (level B)	4.20 ± 0.79, 0.19	4.20 ± 0.63, 0.15
② Abdominal breathing	4.20 ± 0.79, 0.19	Merged
③ Following the audio guides (level B)	4.30 ± 0.67, 0.16	4.30 ± 0.68, 0.16
④ 15 minutes at a time, twice a week (level B)	4.40 ± 0.52, 0.12	4.20 ± 0.42, 0.10
2.5. Soothing music (level A)	4.10 ± 0.32, 0.08	Merged
① Western soothing music	3.80 ± 0.92, 0.24	Merged
② Chinese soothing music	3.90 ± 0.99, 0.25	Merged
2.6 Physical exercise	4.40 ± 0.70, 0.16	4.50 ± 0.71, 0.16
(1) Patients without exercise contraindication are given a personalized, medium or low intensity of aerobics and progressive resistance exercise for 30 to 60 minutes a day (level A)	-	4.50 ± 0.71, 0.16
① 30 to 60 minutes per day	4.10 ± 0.74, 0.18	Merged
② Taichi, Baduanjin	4.20 ± 0.79, 0.19	Merged
③ Health exercises	4.40 ± 0.70, 0.16	Merged
④ Resistance training	4.20 ± 0.79, 0.19	Merged
2.7. Color emotion management (level C)	3.90 ± 0.99, 0.25	Deleted
① Color management of interior space	3.60 ± 0.84, 0.23	Deleted
② Health education manual color matching	3.60 ± 1.08, 0.30	Deleted

3 Sleep improving	4.90 ± 0.32, 0.07	Merged
3.1. Sleep behavior adjustment and nursing (level B)	4.80 ± 0.42, 0.09	Merged
① Form good sleeping habits and create a good sleeping environment	4.80 ± 0.42, 0.09	Merged
② Insomnia patients add Chinese medicine sleeping-improving diet according to patient's TCM constitution	3.90 ± 0.88, 0.23	Merged
③ Insomnia patients press hypnotic points before sleeping	3.70 ± 0.95, 0.26	Merged

4. function promoting	4.70 ± 0.48, 0.10	Merged
(2) Doing finger exercises for 3 times a day (level B)∗	4.70 ± 0.48, 0.10	4.60 ± 0.52, 0.11
① 10 sections, each section repeats 8 eight-beats (level B)∗	4.40 ± 0.70, 0.16	4.50 ± 0.53, 0.12
(3) Patients with dysphagia are given feeding training to improve their ability to feed (level B)∗	4.70 ± 0.48, 0.10	4.70 ± 0.48, 0.10
① Strengthening swallowing function (level B)∗	4.60 ± 0.52, 0.11	4.70 ± 0.48, 0.10
② Reducing food residue (level B)∗	4.50 ± 0.53, 0.12	4.70 ± 0.48, 0.10
③ Preventing aspiration (level B)∗	4.80 ± 0.42, 0.09	4.70 ± 0.48, 0.10
(4) Guiding patients to have recreation rehabilitation, once a day (level B)∗	-	4.60 ± 0.70, 0.15

5. Interaction maintaining	4.80 ± 0.42, 0.09	4.80 ± 0.42, 0.09
(1) Patients and caregivers are encouraged to participate in the formulation and implementation of personalized nursing plans and got a home care service network platform by the electronic network (level B)	4.70 ± 0.48, 0.10	4.70 ± 0.48, 0.10
① Telephone follow-up once a month (level C)	4.20 ± 0.79, 0.19	4.50 ± 0.53, 0.12
② Getting professional guidance once a week through social software (level C)	4.00 ± 0.82, 0.20	4.40 ± 0.52, 0.12
③ Home visits per month	4.20 ± 1.23, 0.29	Deleted

Some merged items in Round 2 had no corresponding scores in Round 1. ^*∗*^ Belonging to “physical Exercise” in Round 2.

## Data Availability

The data used to support the findings of this study are available from the first author upon request.
